# Abnormal systolic and diastolic LV motion by novel tissue phase mapping accounts for functional capacity in pulmonary hypertension

**DOI:** 10.1186/1532-429X-16-S1-P253

**Published:** 2014-01-16

**Authors:** Daniel S Knight, Jennifer A Steeden, John G Coghlan, Andrew Taylor, Vivek Muthurangu

**Affiliations:** 1 UCL Centre for Cardiovascular Imaging, University College London, London, UK; 2 Division of medicine, University College London, Royal Free Campus, London, UK

## Background

Pulmonary arterial hypertension (PAH) is characterized by RV dysfunction. However, abnormal ventricular interactions also result in abnormalities of systolic and diastolic LV function. Unfortunately, these changes may be subtle and are often overlooked in clinical assessment. In this study, a novel self-gated tissue phase mapping (TPM) sequence was used to evaluate the components of LV motion in patients with PAH. The aim was to better characterize abnormal LV function and to assess its functional significance.

## Methods

The study population consisted of 20 patients with PAH and 20 age- and sex-matched healthy volunteers. All patients underwent a 6-minute walk and NT-proBNP testing, and 12 patients underwent right heart catheterization within 1 calendar month of the CMR. A self-navigated retrospectively gated golden-angle spiral sequence was used to perform TPM of basal- and mid-ventricular slices. The raw TPM data was used to calculate radial, tangential (twist) and longitudinal myocardial velocities for the two LV slices. In addition, all patients underwent conventional LV and RV volumetric assessment.

## Results

**Figure 1:**
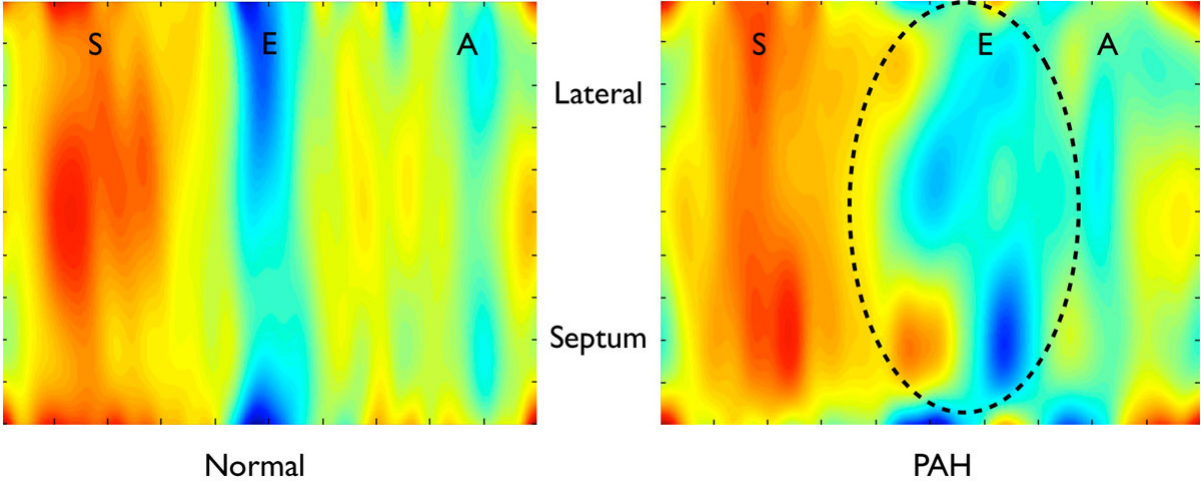
**Colour-maps displaying radial velocities against time for a mid LV short-axis slice in a healthy volunteer and in a patient with PAH during a single cardiac cycle**. Note the abnormal septal motion in PAH, with consequent discordant segmental early diastolic radial LV motion that progresses from the septum towards the lateral LV wall (circled).

The LVEDV was lower in PAH with preserved ejection fraction compared to normal controls. TPM revealed significant abnormalities of systolic and diastolic motion in both the basal and mid LV cavities of patients with PAH (table 1 Figure 1). Specifically, there was a reduction in systolic twist and diastolic radial and longitudinal expansion. Furthermore, peak LV circumferential untwisting was delayed in PAH (65.1 ± 46 ms after radial e-wave). Interestingly, LV metrics by TPM did not correlate with PA pressure. However, on univariate analysis, peak LV tangential (r = 0.504, p = 0.033) and radial (r = 0.454, p = 0.044) s-waves, and LV radial e-waves (r = 0.748, p = 0.00023) did correlate with 6-minute walk distance in PAH patients, as did LVEF and LVSV (but not invasive measures). Nevertheless, on multivariate analysis, only mid-LV peak radial e-wave velocity was an independent predictor of 6-minute walk distance (β = 0.844, p < 0.0006). In addition, peak LV radial e- (r = -0.645, p = 0.004) and a-waves (r = 0.569, p = 0.014) also correlated with NT-proBNP levels in PAH.

**Table 1:** Table of LV volumes, ejection fractions and TPM velocities in PAH and healthy controls

LV mid-cavity peak velocities (cm/s)	PAH	Healthy volunteers	P
Radial S	2.22 ± 0.42	2.50 ± 0.38	< 0.04

Tangential S	0.53 ± 0.70	1.18 ± 0.83	< 0.02

Longitudinal S	3.34 ± 1.68	3.55 ± 0.93	0.63

Radial E	2.43 ± 1.13	3.10 ± 0.80	< 0.04

Tangential E	1.77 ± 0.40	0.61 ± 0.48	< 9 × 10^-10^

Longitudinal E	2.83 ± 1.33	4.56 ± 1.32	< 0.0003

Radial A	1.66 ± 0.83	1.57 ± 0.44	0.68

Tangential A	0.60 ± 1.07	0.59 ± 0.73	0.95

Longitudinal A	2.37 ± 1.14	2.55 ± 0.84	0.58

LVEDV (mL)	88.9 ± 29.7	125.5 ± 25.7	< 0.0002

LVESV (mL)	33.0 ± 17.6	42.7 ± 15.2	0.07

LVEF (%)	64.1 ± 12.0	66.4 ± 8.2	0.48

## Conclusions

We have shown using a novel TPM sequence that there are significant abnormalities of LV systolic tangential and diastolic radial and longitudinal velocities in PAH. Furthermore, we have demonstrated that there is temporal distortion of the separate components of early diastolic LV motion. Importantly, we have shown that these abnormal measures correlate with 6-minute walk distances, implying that part of reduced exercise tolerance in PAH is secondary to LV disease. In fact, the only independent predictor of the 6-minute walk test was the radial e-wave, which suggests that LV early diastolic dysfunction may play a pivotal role in the disease symptoms.

## Funding

Drs. Daniel Knight and Vivek Muthurangu are funded by the British Heart Foundation (BHF).

